# Implementing civic engagement within mental health services in South East Asia: a systematic review and realist synthesis of current evidence

**DOI:** 10.1186/s13033-020-00352-z

**Published:** 2020-03-10

**Authors:** Karen James, Helen Brooks, Herni Susanti, Jessica Waddingham, Irman Irmansyah, Budi-Anna Keliat, Bagus Utomo, Diana Rose, Erminia Colucci, Karina Lovell

**Affiliations:** 1 Centre for Health and Social Care Research, Faculty of Health, Social Care and Education, Kingston and St Georges, 6th Floor Hunter Wing, Cranmer Terrace, London, UK; 2 Department of Health Services Research, Institute of Population Health Sciences, University of Liverpool, Liverpool, UK; 3 Faculty of Nursing, University of Indonesia, Depok, Indonesia; 4 University of Nottingham, Nottingham, UK; 5 National Institute of Health Research and Development, Jakarta, Indonesia; 6 Marzoeki Mahdi Hospital, Bogor, Indonesia; 7 Komunitas Peduli Skizofrenia Indonesia, Jakarta, Indonesia; 8 Department of Health Services Research, Kings College London, London, UK; 9 Department of Psychology, Middlesex University, London, UK; 10 Division of Nursing, Midwifery and Social Work, School of Health Sciences, Faculty of Biology, Medicine and Health, University of Manchester, Manchester Academic Health Science Centre, Manchester, UK; 11 Greater Manchester Mental Health NHS Foundation Trust, Manchester, UK

**Keywords:** Civic engagement, Patient and public involvement, Mental health, South East Asia, Realist synthesis, Global health

## Abstract

**Introduction:**

Civic engagement (CE) has the potential to transform mental health services and could be particularly important for low and middle-income countries (LMICs), which are rapidly developing to respond to the burden of poor mental health. Research from high income countries has found many challenges associated with the meaningful implementation of CE in practice, but this has been underexplored in LIMCS and in South East Asia (SEA) in particular.

**Methods:**

We completed a realist synthesis and systematic review of peer reviewed publications and grey literature to identify the context and actions which promote successful implementation of CE approaches in SEA. We used a theory-driven approach—realist synthesis—to analyse data and develop context-mechanism-outcome configurations that can be used to explain how civic engagement approaches operate in South East Asian contexts. We worked closely with patient and public representatives to guide the review from the outset.

**Results:**

Fifty-seven published and unpublished articles were included, 24 were evaluations of CE, including two Randomized Controlled Trials. The majority of CE interventions featured uptake or adaptation of Western models of care. We identified important cultural differences in the enactment of civic engagement in SEA contexts and four mechanisms which, alongside their contextual barriers and facilitators, can be used to explain how civic engagement produces a range of outcomes for people experiencing mental health problems, their families and communities. Our review illustrates how CE interventions can be successfully implemented in SEA, however Western models should be adapted to fit with local cultures and values to promote successful implementation. Barriers to implementation included distrust of services/outside agencies, stigma, paternalistic cultures, limited resource and infrastructure.

**Conclusion:**

Our findings provide guidance for the implementation of CE approaches within SEA contexts and identify areas for further research. Due to the collectivist nature of many SEA cultures, and the impact of shared traumas on community mental health, CE might best be implemented at community level, with a focus on relational decision making.

*Registration* This review is registered on PROSPERO: CRD42018087841.

## Background

Civic engagement (CE) is a process through which people become actively and genuinely involved in the planning, development and delivery of services, and in taking action to bring about change [1]. In a health systems context this is often known as ‘patient and public involvement’. Civic engagement has the potential to transform mental health systems, and when successfully implemented its benefits include improved access to and quality of care, reduced stigma, increased health literacy, social inclusion, better outcomes for service users, improved staff attitudes and reduced service costs [2–6].

In high income countries, the involvement of people with experience of mental health problems in the design and delivery of services now forms a central part of modern mental health research, policy and guidance [7, 8], and CE is a key focus of the WHO strategy for strengthening health systems globally [2]. It is particularly important for mental health systems in low and middle-income countries (LMICs), which are rapidly developing to respond to the substantial burden of mental health difficulties. The rights of people with mental health problems to be involved as equals in decisions about their care are guaranteed by the Convention on the Rights of Persons with Disabilities, now ratified by over 150 countries, however a wide range of violations have been reported in LMICs [9]. Stigma towards those with mental health problems is pervasive in LMICs which represents a significant barrier to the implementation of civic engagement activities [10–12]. Such vulnerabilities to human rights abuse and stigma towards those with mental health problems have also been reported in Western contexts [13].

South East Asia is a sub region of Asia made up of 11 diverse countries between the Indian and Pacific Ocean. Mental health resources vary between countries but generally speaking mental health has been a low priority across the region with treatment gaps exceeding 90% in some countries [14]. Factors affecting the delivery of mental health services include poverty, inequality, rapid urbanization, stigma, lack of investment in mental health, insufficient legislative infrastructure and periods of intense social and cultural change [12, 14].

An examination of existing evidence highlights the potential utility of civic engagement for South East Asian populations. One commonly used component of CE is service user involvement in mental health care planning, often referred to as shared-decision making (SDM). There is a general consensus amongst all stakeholders about the value of such initiatives, although discussions continue [8, 15]. This form of CE has been shown to enhance mental health literacy and increase confidence amongst those who use services which can result in improved health outcomes [5, 16]. It can also lead to improved information about, and access to, mental health care [3], as well as enhancing relationships between patients and clinicians [17]. In some cases SDM has been shown to improve satisfaction amongst service users and enhance systemic performance brought about by increased accountability and more patient focused services [18].

Such findings are however not ubiquitous, demonstrating the complexity of CE implementation. For example, a recent systematic review identified 11 studies which evaluated a range of interventions designed to improve shared decision making for people with psychosis and identified some evidence of impact on the ‘subjective empowerment’ of service users [19]. However, included studies were small scale and of modest quality. Others demonstrate evidence of improvements in affective-cognitive outcomes but find insufficient evidence to support behavioural or health outcomes [20, 21].

Involving people with personal experience of mental health problems in the delivery of services is another common form of civic engagement within mental health systems, often known as peer support. This can include mutual support groups, one to one support delivered by a person with experience of mental health problems (a ‘peer’) and peer-led (i.e. managed) mental health services [22]. Findings regarding the effectiveness of peer support have been mixed. Several Randomised Controlled Trials have found some evidence that peer support led to significant reductions in symptom severity and had a positive impact on personal recovery, hope and empowerment [23–26]. However, systematic reviews and meta-analyses report that, on the whole, there is a lack of high quality research and a large amount of heterogeneity between studies and models of peer support delivered, which makes it difficult to draw any firm conclusions about the effectiveness of peer support [22, 27].

Although current evidence is limited, civic engagement within mental health services shows some promise, and it is important to note that the value of user involvement is not limited to improving the quality of care; involvement in healthcare is often viewed as a democratic or ethical requirement of good practice in mental health services, reflecting the moral right to self-determination [3, 8]. Civic engagement therefore lies at the intersection of evidence-based and values-based practice, and its values are central to contemporary global mental health policy.

Research in English speaking countries has found many challenges associated with the meaningful implementation of CE in routine practice, which may account for the limited translation of CE polices into demonstrable impact on service and patient level outcomes. These include a lack of accessible information for people about mental health and the rights of service users, a lack of awareness of involvement amongst service providers, variations in understanding of and commitment to, involvement amongst service users and professionals, increased costs, concerns about risk and ‘representativeness’, and resistance amongst professionals and organisations to collaborative ways of working [6, 20, 28, 29]. This is an underexplored area in LMICS, and in South East Asia (SEA) in particular, however there are likely to be unique challenges (e.g. resource limitations, lack of evidence about how best to involve people in the design and delivery of mental health services and high levels of stigma) to the meaningful implementation of CE within these contexts [30].

This review aimed to (i) identify the range of approaches to civic engagement in mental health services implemented in SEA and (ii) synthesize current evidence around the context, mechanisms and outcomes of these approaches. Research questions were:

1. What types of civic engagement approaches have been implemented in mental health services in SEA?
2. What are the mechanisms through which civic engagement interventions are expected to affect individual, system and community level mental health outcomes in SEA?
3. What contextual factors act as barriers to, or facilitators of successful implementation of CE in SEA?

The study was designed in collaboration with people in SEA with personal experience of mental health problems, or of caring for a loved one with a psychiatric diagnosis, including members of the peer-led organisation, Komunitas Peduli Skizofrenia Indonesia (KPSI). An advisory group consisting of 12 people who either had lived experience of psychosis or cared for someone with a diagnosis of psychosis were recruited as part of a wider study exploring the potential of involving patients, carers and communities to strengthen mental health systems in Indonesia [31]. The group and PPI co-applicant Utomo were consulted on the search terms, grey literature searching and interpretation of data from included studies.

## Methods

We completed a realist synthesis of peer reviewed publications and grey literature reporting civic engagement approaches in SEA, guided by the RAMESES quality standards for realist synthesis [32]. Realist synthesis aims to discover “what works for whom, under what circumstances, how and why?” by exploring interactions between context, mechanisms of action and outcomes of an intervention [33]. This was our chosen approach because (i) it is particularly useful when evaluating complex interventions, such as civic engagement, which are likely to work in different ways when implemented in different settings (ii) it allows for the synthesis of a diverse range of data sources (as CE in SEA is an under researched area we wanted to capture all available evidence), and (iii) it elicits detailed and practical information, which can be used by policy makers, managers and service users in the planning and implementation of programmes.

### Scoping the literature

**Fig. 1:**
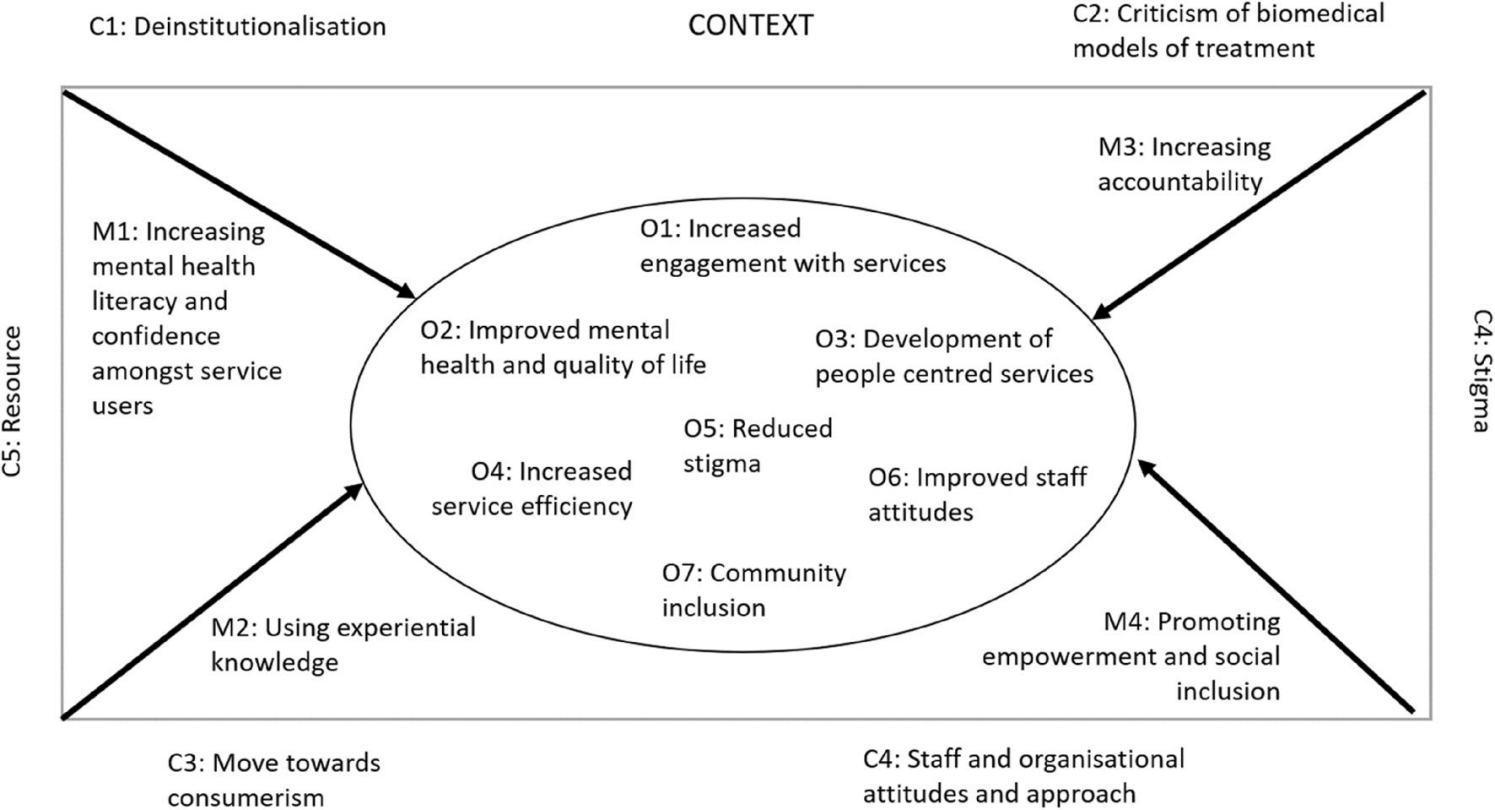
Initial programme theory

The first step of a realist synthesis is to identify the underlying assumptions about how an intervention is thought to work, which are then tested and developed through a review of the available evidence [32]. Following a scoping review of the literature, we developed an initial program theory, outlined in Fig. 1, in-line with other realist reviews [34], which identified the key components of civic engagement (see [31] for a detailed description and overview of relevant literature). This was presented to our study management and PPI advisory group consisting of academics, clinicians and people with lived experience (in the UK and Indonesia), and refined based on their feedback.

### Search strategy and selection criteria

We adopted the WHO definition, which describes civic engagement as a process by which people are enabled to become actively and genuinely involved in planning, developing and delivering services and in taking action to bring about change [1]. For this study we included any data reporting the involvement of lay people (i.e. community members, service users and carers) in the design and delivery of mental health services.

Inclusion criteria were sources reporting:

i. Civic engagement.
ii. Within mental health services (including primary care, and third sector services, if the project addressed mental health or psychological wellbeing).
iii. In South East Asia (Brunei, Burma, Cambodia, Timor-Leste, Indonesia, Laos, Malaysia, Philippines, Singapore, Thailand, Vietnam).

Exclusion criteria were sources:

iv. Not accessible online or via inter-library loan.
v. Published in abstract only form with insufficient detail to extract relevant information.
vi. Not in Bahasa or English.

Search terms (Additional file 1) were developed drawing on literature reviews and other key publications in the field, and in consultation with the project team. Systematic electronic database searches were conducted in May 2018 from the earliest record using ASSIA, Embase, International Bibliography of the Social Sciences, Medline, PsychInfo, Social Science Full Text, Sociological Abstracts, and Web of Science. Grey literature searches incorporated i) Google searches using key search terms (first 10 pages were screened), ii) searches of grey literature databases and university repositories iii) searches of target websites, identified by the research team and advisory group iv) consultation with three experts who were contacted via email and asked to identify any potentially relevant data sources (see Additional file 2 for more information).

Screening was completed using the data management software Covidence (http://www.covidence.org). For peer reviewed publications, titles and abstracts were double-screened for eligibility, and for grey literature, abstracts, executive summaries or table of contents were screened. Screening was completed by HB, KJ, KL and JW. Any conflicts were resolved by an independent reviewer. Full texts were screened for inclusion by HB and KJ independently and conflicts were resolved through discussion between authors until a consensus was reached.

### Data quality

In line with realist review guidelines, included articles were assessed for their relevance and methodological rigour [32]. In this context, relevance related to the identification of evidence to develop the initial program theory and articles were not excluded based on methodological type (e.g. unpublished works) or quality. In some papers, particularly editorials, it was not evident whether assertions were based on empirical data or solely on author opinion. In these cases, content was used in conjunction with other empirical data to support interpretations and to build explanatory CMO configurations. Included articles are described in Additional file 3. Data quality is discussed within the presentation of findings.

### Data extraction and analysis

Data were extracted into an Excel database which included fields for study information, features of the intervention, and context, mechanisms of action and outcomes at a micro (between individuals), meso (service or community) and macro (national) level across the health system. CMO configurations were developed by searching for themes across the data through an iterative process of discussion between HB and KJ. Draft configurations were presented to our advisory group and further developed based on their feedback.

## Results

### Characteristics of the data

**Fig. 2:**
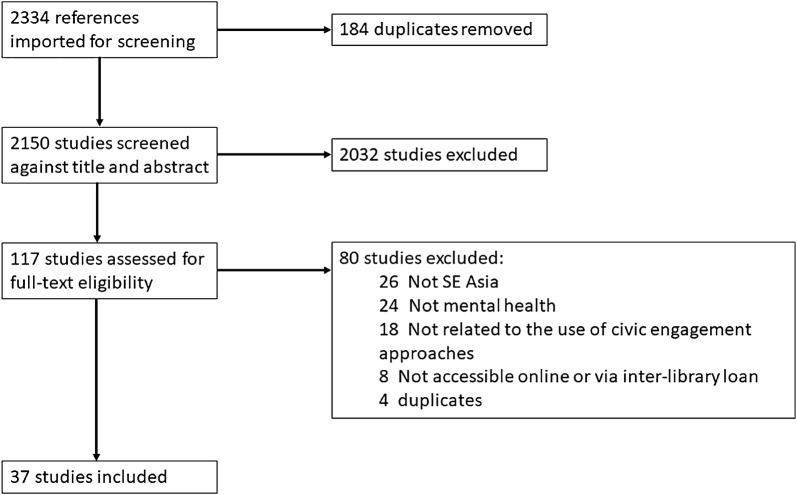
Flow diagram of peer reviewed publication retrieval, adapted from the Preferred Reporting Items for Systematic Review and Metaanalyses (PRISMA) flow diagram

Grey literature searches identified 20 publications. The retrieval process for peer reviewed publications is outlined in Fig. 2. Table 1 gives an overview of the different types of data sources included. The 57 publications included in the review comprised 33 journal articles, 12 theses or dissertations, four reports, four articles, three conference abstracts, and one book chapter.

**Table 1:** Description of data included in the review

Type of data	Studies and methods used
*n* = 24—Evaluation (or evaluation protocol) of a program, intervention or health system featuring CE	Randomised Controlled Trial [35–38]; Pre/post evaluation [43, 52, 53, 72, 83–86]; Survey [54, 62, 63]; Mixed methods [44, 45, 47, 49, 87, 88]; Qualitative interviews and/or focus groups [40, 73]; Study protocol [42]
*n* = 10—Primary (exploratory) research with a focus on/findings relating to CE	Qualitative interviews and/or focus groups [42, 55, 56, 60, 89–91]; Ethnography [61]; Survey [67]; Mixed methods [51]
*n* = 7—Description of the features and implementation of a specific program or intervention incorporating elements of CE	Publications [41, 46, 48, 64, 70, 92, 93]
*n* = 4—Narrative review of, or commentary on, models of care featuring CE (e.g. Shared Decision Making, Person Centered Care)	Publications [39, 57, 59, 94]
*n* = 12—Overview or review of countrywide mental health policy, legislation or systems, featuring elements or incorporating discussion of CE	Publications [50, 58, 65, 66, 68, 69, 71, 74, 95–98]

Most publications were from Indonesia, followed by Singapore and Thailand (Table 2). Just half (50%) of peer reviewed publications featured first authors based in SEA, and on average the majority (57%) of co-authors were based in Western countries.

**Table 2:** Number of publications by country

Country	*n*	%
Indonesia	25	44
Singapore	8	14
Thailand	7	12
Cambodia	4	7
Malaysia	4	7
South East Asia	4	7
Philippines	2	4
Lao	1	2
Myanmar	1	2
Vietnam	1	2

Twenty-four publications reported data from direct evaluations of CE (Table 1) and only two studies (reported in four publications) used Randomised Controlled Trials; in Singapore a peer-led self-management programme led to significant improvements in participant empowerment, perceived recovery, social support and symptom severity, as measured by client and professional ratings [35, 36]. However, these data are reported as part of a series of conference proceedings and it is therefore difficult to fully assess the quality of the research. A classroom-based intervention in the post conflict area of Poso, Indonesia, moderately reduced PTSD symptoms for girls and maintained hope for both genders but had no effect on depression or anxiety [37, 38].

### Features of civic engagement

Where interventions or approaches incorporating elements of civic engagement were described, the majority featured uptake or adaptation of Western models of care, such as shared decision making [39], early intervention for psychosis services, including elements of joint care planning or shared decision making [40, 41], or peer led self-management programs [35, 36, 42]. Other interventions were developed by clinicians or other professionals based in South East Asia [43–46], or by members of the local community itself [46–48].

There were important cultural differences identified in relation to the enactment of civic engagement in SEA contexts. Most sources described civic engagement as featuring people with a lived experience of mental health problems (n = 18) or family members/carers (n = 18), however members of the wider local community (n = 15) were also frequently involved. Community members were typically identified as ‘trusted individuals’, or community leaders, such as village chiefs, elders, teachers, or religious figures [37, 46, 49, 50]. Civic engagement activity mainly comprised the involvement of service users, carers, or community members in the delivery of services (n = 18), other sources described the involvement of service users, or their families in decision making about care (n = 10), or in the development or adaptation of services (n = 6). One featured indirect involvement, where research with service users and carers was used to inform the development of an intervention [51].

### Final programme theory

**Fig. 3:**
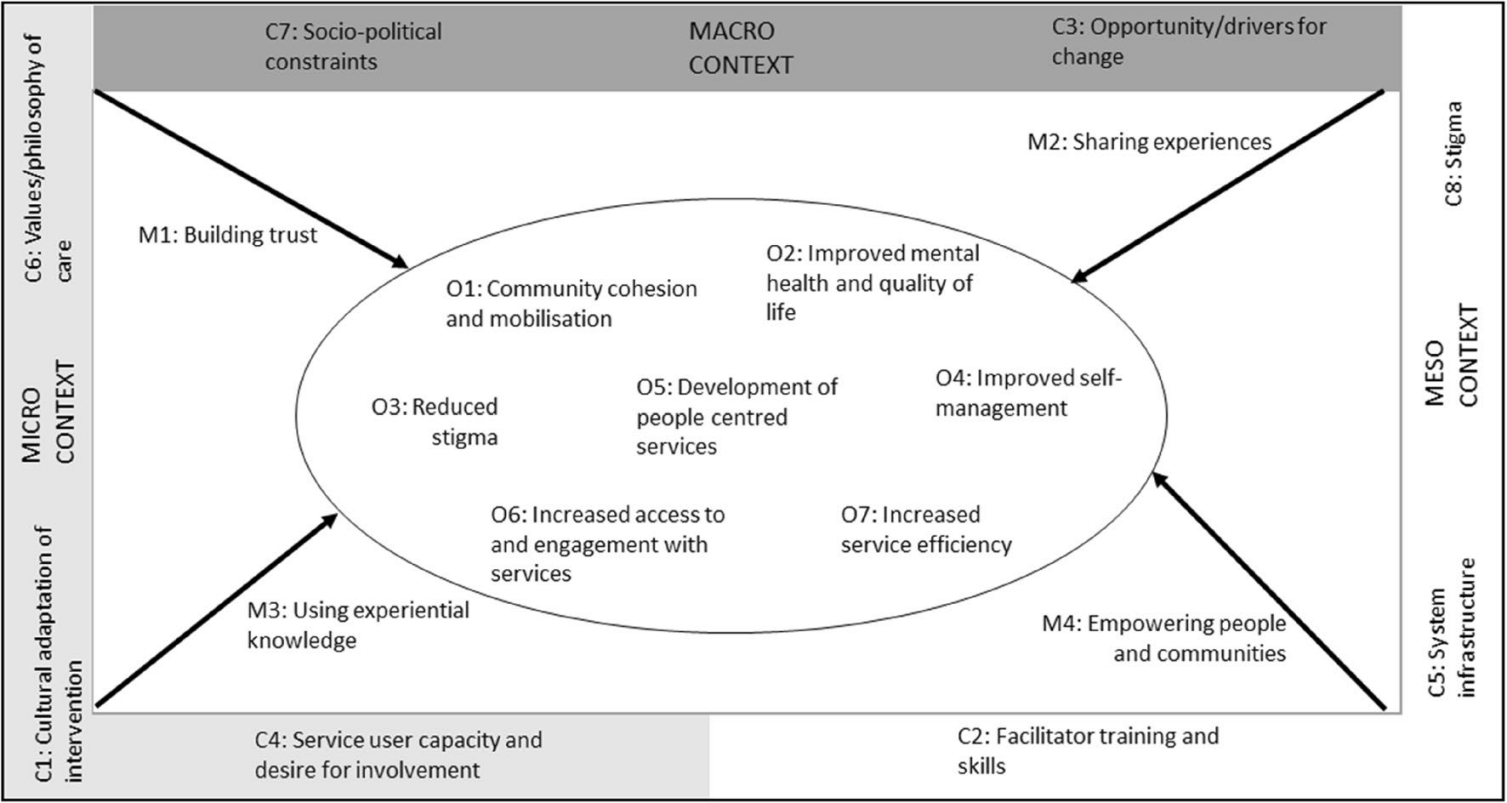
Overview of final programme theory

We identified important cultural differences in relation to the enactment of civic engagement in SEA contexts and four mechanisms which, alongside their contextual barriers and facilitators, can be used to explain how civic engagement produces a range of outcomes for people experiencing mental health problems, their families and communities in SEA. These are described in detail below, and summarised in Fig. 3, which gives an overview of our final programme theory. Although we found further evidence for a number of the outcomes identified in the initial scoping exercise (Fig. 1), many of the contextual barriers and facilitators to implementation of CE changed following the review. These operated at multiple levels across the system, and are identified in Fig. 3 as acting at a micro (between individuals), meso (health system or community) and macro (national) level.

### Description of CMO configurations

#### Building trust

*CMO 1: Effective civic engagement means developing trusting relationships between clinicians, service users and their families, and also amongst members of a community (M1), which in turn can promote community cohesion (O5), the development of person centered care (O5) and increased access to and engagement with services (O6). This process can be difficult in communities which have experienced conflict or trauma (C7), where there is an inherent distrust of services (C4) or where services are resistant to working collaboratively with service users (C6).*

There was some evidence of how, at a group level, building trust (M1) could help to build more cohesive communities (O1), particularly amongst family members [42, 52–55], and within communities that had been impacted by traumas, such as armed conflict, political suppression or natural disasters, leading to a sense of fear and a collective loss of meaning and social structure (C7 [48]). However, in these cultural contexts building trust was a challenge [48, 50, 56, 57] and a number of initiatives made efforts to engage with the local community many months before the project began, for example, by arranging community entertainment or discussions and talks [46]. Some sources reported an inherent distrust of ‘modern’ mental health services, which people associated with ‘colonial expansion’ (C7) because they had adopted Western models of care. These services also disregarded traditional therapeutic practices, which further alienated local communities [58].

At an individual level, building trust could be difficult when service users and their families did not want to work with services (C4) due to negative experiences of care, or relationship conflict [44, 55]. However, trusting relationships between services, service users and their communities could also lead to increased engagement and service use (O6 [42, 44, 59]), this was particularly evident where programs trained trusted community members (kader) to work alongside clinicians, allowing them access to communities that might otherwise not be engaged [49, 53]. Improved relationships between service users, families and clinicians also meant they could work more collaboratively, increasing the likelihood of person centered care (O5). However, this was unlikely to happen within services which adopted a paternalistic approach (C6), where compliance was expected, and forms of containment, such as locked wards, were used in a punitive fashion [59–61].

#### Sharing experiences

*CMO 2: Civic engagement requires people to share their experiences of mental health challenges in order to improve their local services (M2). Where initiatives are adapted to enable all community members to contribute (C1), and where there is skilled staff to facilitate (C2), this process can help to reduce stigma (O1), promote community cohesion (O1) and improve mental health and quality of life (O2).*

Sharing experiences amongst community members (M2) provided an opportunity for people to help one another and develop a network of support which reduced social isolation and helped them to manage their mental health outside of services (O2 [46]). There was evidence that this process fostered community cohesion (O1), particularly in projects which encouraged communities to develop a shared understanding of collective experiences, such as displacement and conflict between different ethnic or religious groups [48]. It was important that project workers had the necessary skills, experience and support from senior members to staff to enable them to facilitate these discussions (C2 [46, 62]). Some projects had to be adapted to allow all members to contribute, for example, within some communities where male voices and those of community leaders would dominate, it was necessary to have women only groups so that women’s voices could be heard (C1 [48]).

The pervasive stigma associated with a psychiatric diagnosis in SEA, and particularly beliefs that mental illness is a supernatural occurrence, prevented some people from disclosing their mental health challenges (C8). Where Western models of peer support were implemented, service users questioned why the trainer openly discussed their mental health, as this was rarely talked about within their culture [63]. However, there was also evidence that openly discussing and sharing experiences of poor mental health and involving local people in the development of services could reduce stigma and promote social inclusion (O3 [35, 36, 42, 47, 48]), particularly when people rejoined their community after being discharged from inpatient services [64].

#### Using experiential knowledge

*CMO 3: Through the process of civic engagement, experiential knowledge, such as personal experience of mental health problems, or knowledge of the local community, is used to inform the design and delivery of local services (M3), leading to person centered care (O5), increased access and engagement with services (O6), increased service efficiency (O7) and improved mental health and quality of life (O2).*

Including experiential knowledge in the development of services meant that service providers developed a greater understanding of local mental health needs, and how interventions could best be designed to meet these needs. There was evidence that this could lead to more person centered care (O5 [44, 48, 51, 59]), increased service use (O6 [44, 50, 59]) and efficiency (O7 [41, 42, 44, 59, 64, 65]) and improved mental health and quality of life of service users (O2 [41–43, 47, 59, 66]). Some sources also reported how this helped to reduce the stigma associated with poor mental health (O3 [48, 64, 67]), particularly in peer-led projects, such as a Thai psychoeducation intervention where the trainers had personal experience of caring for family members with a serious mental illness [63].

This often required a fundamental shift in current ways of working; a number of sources described how key opportunities for change (C3) opened up space for lived experience to inform the design and delivery of new services. For example, following the tsunami in Indonesia there was an influx of donations and interest from international agencies, which led to the development of a new, decentralised, mental healthcare system, staffed at a village level by community volunteers (kader [49]). There were also accounts of how campaigns and pressure from local NGOs and international agencies, such as the World Health Organisation, were drivers for change (O3 [58, 68]). A number of sources described how globalisation and advances in communication meant that countries became interested in, and influenced by, Western models of care (O3), such as peer support and self-management programs, research demonstrating the effectiveness of these ways of working in Western countries, and also social movements, such as the recovery movement (now an intervention) [39, 42, 59]. However, these models were often at odds with local practices (C6), for example, whilst there was interest in the shared decision making model in Malaysia, there were concerns that because of its focus on the needs of the individual it may not ‘work’ within Malaysian culture, as people are strongly influenced by their families and communities [39]. A review of person centered care in Indonesia described how this approach challenges social norms; for example because health-care professionals are highly respected and often come from a higher class of society than service users, compliance is often assumed, and people do not expect to be involved in decisions related to their care [59].

A lack of understanding of mental health and the benefits of involvement, awareness of patient’s rights and a lack of confidence and knowledge about how to assert these rights was a barrier (C4 [39, 44, 46, 64, 69]). A further barrier was the limited infrastructure available to support involvement (C5), such as limited funds, transportation to access remote communities, understaffing, and limited organisational support for involvement from NGOs and statutory services, at both a local and national level [39, 47, 49, 58, 59, 62, 69].

#### Empowering individuals and communities

*CMO 4: Civic engagement activities may generate increased community cohesion and mobilisation (O1), engagement with services (07) and mental health (02) and reduced stigma (O3) where these initiatives are able to instill feelings of empowerment and hope amongst recipients (M4). Empowerment is most likely to be triggered in contexts where all stakeholders value civic engagement (C1) and health services provide the infrastructure for empowerment practice (C5).*

Civic engagement approaches rely on the process of individual, community and political empowerment. Included studies demonstrated how such approaches could provide normalisation and validation of people’s experiences in South East Asian populations [70]. Interventions that were able to equip service users, family members and communities with knowledge and skills about mental health empowered service users to take responsibility for managing their own illness and gave family members and communities the confidence to support service users better [40, 65]. As a result, civic engagement interventions could improve mental health and quality of life for service users and carers (O2 [35, 36, 39, 40, 42, 67, 71]).

The mechanism of empowerment was facilitated by contexts which understood and were supportive of recovery or civic engagement at a micro, meso or macro level (C3/C4 [39, 42, 46, 50]). The collective culture central to some South East Asian countries was an important contextual facilitator [46, 64] but such cultures were not ubiquitous within included publications [50]. Other barriers to empowerment included low mental health literacy and desire for involvement amongst service users and their carers and high levels of stigma amongst communities (C4, C8 [39, 42, 50, 62, 64, 72–74]).

Hope and accountability were identified as central features of empowerment within studies [64, 67]. Hope was particularly important for communities receiving interventions following disasters especially for those who believed strongly in ‘karma’ [46]. Sharing recovery stories was one way in which identified civic engagement activities instilled hope in others by allowing them to see new possibilities and solutions in relation to current mental health difficulties [62, 63]. Accountability to themselves and others was identified as an important part of the process of empowerment within identified interventions [67].

## Discussion

We conducted a realist synthesis of published and grey literature to systematically examine the range of civic engagement approaches which have been used in SEA, and the mechanisms through which civic engagement is thought to bring about individual, system and community level mental health outcomes. The findings of this review add to the current literature on the use of civic engagement in SEA. Although limited in number, Randomised Controlled Trials suggest the potential utility of such approaches for this population and these were supported by rigorous qualitative studies and survey data; however, there are a number of factors which require consideration by health service providers prior to implementation.

Relationships between stakeholders and the development of interpersonal trust were fundamental to the success of civic engagement initiatives which represented a particular cultural challenge within SEA contexts. In line with data from other countries [75], there was evidence that some service users were motivated to engage in civic engagement activities within mental health services; however this was not the case in all studies. For this population, desire for engagement was impeded by past negative experiences with mental health services and concerns/distrust about influence from outside agencies (Western practitioners, NGOs, etc.). This was particularly true in communities with a history of Western influence or interpersonal conflict, and where interventions were not culturally adapted prior to implementation. Reported stigma relating to mental health conditions was pervasive in the literature, as were attributions of mental illness which conflicted with conventional medical orthodoxy which also reduced the desire and capacity to engage with mental health services.

As with all innovation in mental health services and in line with implementation research, support is required from meso and macro levels within the health care system in order to optimise conditions for implementation [76] (Fig. 2). Publications reported contextual factors which mirrored implementation difficulties reported across the world [77], such as the historical use of coercion and control within mental health services, paternalistic cultures, suboptimal infrastructure and resource limitations. Particular cultural factors considered important for SEA populations included involvement of wider communities (considered fundamental to success), geographical disbursement of populations (which impeded service access and delivery) and the role of collectivist cultures.

The utilization of civic engagement in western contexts is not without challenges and similar barriers were identified in studies included in the current review [6, 20, 78]. Implementation challenges relating to empowerment practice in mental health in particular, and the need to develop infrastructure to support such approaches at a local and national level, was identified as a key challenge for health services in SEA [62]. In line with research in Western contexts [75], accountability was seen as a core feature of civic engagement, however included publications described minimal ways in which the rights of people with mental health problems were considered, advocated or legislated within most SEA countries [69].

The literature suggests that CE interventions within SEA could be of particular benefit to communities impacted by armed conflict, natural disasters or political suppression; community empowerment was an important mechanism of action of CE, and community cohesion was a common outcome of CE initiatives reported across the literature [46, 48, 63, 70]. Sharing experiences of poor mental health and developing a shared understanding of collective traumas and conflict brought people together, developed new networks of social support, and had the potential to improve mental health and quality of life. CE also developed people’s understanding of mental health and equipped communities with new knowledge and skills which could be used to meet the needs of local people experiencing mental health problems [40, 65]. These initiatives could be particularly valuable in building community resilience and preparedness for natural disasters. For example, following the 2004 Asian Tsunami, trusted community volunteers played a key role in the delivery of much needed mental health services [49].

Despite the prevalence of CE in global health policy, authors have questioned whether CE approaches, largely developed in the West, and grounded in Western values of autonomy and individualism, can be meaningfully implemented in LMICs [79, 80]. The impact of Western influence and globalisation were apparent within review literature; only half of publication first authors were from SEA, and although some interventions were locally developed, most featured the uptake of Western models such as Early Intervention for Psychosis services, or peer led self-management programs [35, 36, 42]. These interventions need to be adapted to fit with local cultures and values, however, our review illustrates how CE interventions can be successfully implemented in SEA and hold a range of potential benefits for people with mental health problems and their communities. Our findings suggest that due to the collectivist nature of many cultures in SEA, and the impact of shared traumas on community mental health, CE interventions might best be implemented at the community level- i.e. involve all community members rather than exclusively people with a psychiatric diagnosis and their carers, as is more common in Western cultures, and should incorporate relational decision making, which considers the broader social and cultural context in which decisions are made [81]. To maximize chances of success CE interventions should ideally operate at multiple levels across the health system (Fig. 3), for example the involvement of service users and their families in developing policy at a national level as well as in the implementation of local initiatives. Consideration should be given to innovative ways to build research capacity in SEA to ensure CE programmes meet the needs of local populations and are led by local stakeholders [82]. Future civic engagement enactment in SEA should include the development of national resources and guidance to ensure ethical issues (e.g. payment and informed consent) are fully considered.

### Strengths and limitations

To our knowledge, this is the first systematic realist review of civic engagement activities in mental health services in South East Asia. This approach allows for the synthesis of a diverse range of data sources, is well suited to the evaluation of complex interventions and elicits data which can be used to inform the planning and implementation of CE programmes. Abstracts and full texts were double screened. Data from qualitative and quantitative studies were triangulated during analysis to develop the final programme theory.

Only two studies utilised randomised control trials to formally evaluate the impact of civic engagement activities and, grey literature searches yielded limited returns. Given the potential benefits of civic engagement for people with mental health problems in SEA as identified within this review, there is a need for further high quality prospective research to build on these findings and develop an evidence base on which health providers can base future decisions. Our review only included publications in English or Bahasa and so there may be an overrepresentation of data from Indonesia. Finally, traditional or faith-based healing featured very little in the literature despite being an important source of support for metal health in South East Asia.

### Conclusions

Our review illustrates how mental health interventions incorporating elements of civic engagement can be successfully implemented in SEA and hold a range of potential benefits, however Western models need to be adapted to fit with local cultures and values. Barriers to implementation included distrust of services and outside agencies, stigma, paternalistic cultures, and limited resource and infrastructure. Due to the collectivist nature of many SEA cultures, and the impact of shared traumas on community mental health, CE might best be implemented at the community level, with a focus on relational decision making.

## Supplementary information

**Additional file 1.** Search terms; table of search terms used.

**Additional file 2.** Target websites for grey literature searches and databases searched; list of databases searched and table of websites.

**Additional file 3.** Description of included publications; table of included publications.

## Data Availability

A summary of data generated or analysed during this study are included in this published article and its supplementary information files. Full datasets are available from the corresponding author on reasonable request.
