# Supplementary material for: Implementing civic engagement within mental health services in South East Asia: a systematic review and realist synthesis of current evidence
Source: Int J Ment Health Syst. 2020 Mar 10;14:17. doi: 10.1186/s13033-020-00352-z (PMC7063827; doi:10.1186/s13033-020-00352-z)
Supplement: Supplementary file 1 — Additional file 1. Search terms; table of search terms used. [file 13033_2020_352_MOESM1_ESM.docx]

**Additional file 1: Search terms**

(“ = key word search, # = mesh term search)

| **Group 1** | **Group 2** | **Group 3** |
| --- | --- | --- |
| “civic engagement” OR  “user involvement” OR  “Co-design” OR  “people focussed design” OR  “community participation” OR  “volunteer OR  “#involvement” OR  “#participat$” OR  patient participation or  “engagement OR  “co-production” OR  “social accountability” OR  “community empowerment” OR  “consumer” OR  “Lived experience” OR  “First hand” | mental disorders OR  ”Mental difficult$ ”OR  ”Psychiatr$” OR  “mental illness” OR  “mental distress” OR  “mental health” OR  “mental well-being” OR  “emotional well-being” OR  “psychological well-being” OR  “community care” OR  “hospital” OR  “primary care” OR  ”Mental health service$” OR  “health service design” OR  “service delivery” | “South East Asia”  OR “Brunei” OR  “Cambodia” OR  “Indonesia” OR  “Laos” OR  “Malaysia” OR  “Myanmar” OR  “Philippines” OR  “Singapore” OR  “Thailand” OR  “Timor Leste” OR  “East Timor” OR  “Vietnam” OR  “Christmas Island” OR  “Cocos Islands”. |
