# Supplementary material for: Implementing civic engagement within mental health services in South East Asia: a systematic review and realist synthesis of current evidence
Source: Int J Ment Health Syst. 2020 Mar 10;14:17. doi: 10.1186/s13033-020-00352-z (PMC7063827; doi:10.1186/s13033-020-00352-z)
Supplement: Supplementary file 2 — Additional file 2. Target websites for grey literature searches and databases searched; list of databases searched and table of websites. [file 13033_2020_352_MOESM2_ESM.docx]

**Additional file 2: Target websites for grey literature searches and databases searched**

**Databases searched:** <http://www.yankes.kemkes.go.id>; <https://osf.io/rvqw2/>; <http://jks.fikes.unsoed.ac.id>; <http://lib.ui.ac.id/>; https://isnet.or.id

| **#** | **Website name/organization** | **Country** | **Link** |
| --- | --- | --- | --- |
| 1 | Transcultural Psychosocial Organisation | Cambodia | <http://tpocambodia.org/> |
| 2 | World Association for Psychosocial Rehabilitation | Global (based in Philippines) | http://www.wapr.org/ |
| 3 | Suryani Institute for Mental Health | Indonesia | http://www.suryani-institute.com/ |
| 4 | Komunitas Peduli Skizofrenia Indonesia | Indonesia | http://www.skizofrenia.org/ |
| 5 | D'Home Mental Health Association Malaysia | Malaysia | http://d-home.org.my/ |
| 6 | Malaysian Mental Health Association | Malaysia | http://mmha.org.my |
| 7 | Health Equity Initiatives | Malaysia, Myanmar | http://www.healthequityinitiatives.com/ |
| 8 | Philippine Psychiatric Association | Philippines | http://ppa.philpsych.ph/ |
| 9 | Sliver Ribbon | Singapore | http://www.silverribbonsingapore.com/ |
| 10 | Awit Foundation | Philippines | <http://www.foundationawit.net/about.html> |
| 11 | Movement for global mental health | Global | <http://www.globalmentalhealth.org/> |
| 12 | Ashoka | Global (but includes SE Asia) | <https://www.ashoka.org/en-gb/our-network> |
| 13 | Basic Needs | Includes work in Lao and Vietnam | <http://www.basicneeds.org/our-approach/> |
