# Supplementary material for: Implementing civic engagement within mental health services in South East Asia: a systematic review and realist synthesis of current evidence
Source: Int J Ment Health Syst. 2020 Mar 10;14:17. doi: 10.1186/s13033-020-00352-z (PMC7063827; doi:10.1186/s13033-020-00352-z)
Supplement: Supplementary file 3 — Additional file 3. Description of included publications; table of included publications. [file 13033_2020_352_MOESM3_ESM.docx]

**Additional file 3: Description of included publications**

| **Reference** | **Country** | **Data type** | **Data source** | **Publication type** | **Method of data collection** | **Description of intervention (actions relating to civic engagement)** |
| --- | --- | --- | --- | --- | --- | --- |
| Ansarias et al. 2007 | Philippines | Peer reviewed literature | Journal article | Description of the features and implementation of a specific program or intervention incorporating elements of CE | N/A | NGO that offers psychosocial and development programmes to individuals traumatized by displacement and armed conflict. Incorporates participatory action research and involving community in research. Trains teachers to facilitate therapeutic play for children in distress. Mass community based interventions which emphasize community participation, empowerment and healing. Community volunteers for medical missions. |
| Gero, 2017 | Indonesia | Grey literature | Article | Evaluation (or evaluation protocol) of a program, intervention or health system featuring CE | Mixed methods | Involvement of family members. Includes games, spiritual activity, working simulation, communication therapy, sharing, guide imagery, music therapy, touching, and humour. |
| Boothby et al. 2011 | Indonesia | Peer reviewed literature | Journal article | Evaluation (or evaluation protocol) of a program, intervention or health system featuring CE | Mixed methods | A new, decentralised mental healthcare system, providing care from household-to-hospital. Previously there was no provision at the district and sub-district levels (i.e. no community support). New system included four puskesmas (community health centers) staffed by community mental health nurses; and at a village level volunteer mental health cadres (from local community) who are trained to identify people in need and refer them to the CMHNs. Cadres role also included raising awareness, reducing stigma and offering outreach support (via home visits) support families. |
| Chan & Rowe 2014 | Thailand | Peer reviewed literature | Journal article | Evaluation (or evaluation protocol) of a program, intervention or health system featuring CE | Survey | A 3 day workshop in assertive communication training, which aimed to build skills for self-management of stress associated with living with/caring for someone with mental illness. The (Australian) trainer had lived experience which was openly and intentionally discussed during the sessions. |
| Chan et al. 2014 | Singapore | Peer reviewed literature | Journal article | Evaluation (or evaluation protocol) of a program, intervention or health system featuring CE | Study protocol | A peer led self-management programme, brief (6-8 weeks) series of weekly meetings with a peer worker (paid) focussed on goal-setting and coping with challenges. Programme based on those implemented in USA and elsewhere. Programme manual reviewed by expert panel including 2 expert patients. Peer workers attended 6 day training workshop. |
| Chanpattana. 2010 | Thailand | Peer reviewed literature | Journal article | Overview or review of countrywide mental health policy, legislation or systems, featuring elements or incorporating discussion of CE | N/A | Current mental health plan aims to improve quality of life by encouraging community participation in relieving mental health problems. |
| Cheausuwantavee. 2005 | Thailand | Peer reviewed literature | Journal article | Description of the features and implementation of a specific program or intervention incorporating elements of CE | N/A | Community Based Rehabilitation (CBR): Participation of people with disabilities in CBR, or strengthening of disabled people’s organisations (DPOs), has been emphasised as the vital part of sustainable CBR. |
| Chiu et al. 2011 | Thailand | Peer reviewed literature | Journal article | Evaluation (or evaluation protocol) of a program, intervention or health system featuring CE | Survey | Family Link Education Programme (FLEP): A peer-led psycho-education intervention for carers, designed by a social worker, service user and carer. Delivered by trainers who were themselves family members of people with SMI. Comprised 8 weekly classes covering symptom recognition and treatment, crisis intervention, rehabilitation and services, communication skills, handling psychological burdens and advocacy. FLEP adopted a peer-led, interactive and empowerment approach that aimed to untangle participants from guilt and emotions while improving knowledge, self-efficacy, family coping and advocacy awareness. |
| Chong et al. 2004 | Singapore | Peer reviewed literature | Journal article | Description of the features and implementation of a specific program or intervention incorporating elements of CE | N/A | An Early Intervention for Psychosis programme. Aims include reducing stigma, improving quality of life for people with psychosis and reducing burden on caregivers. Intervention involves educating general public and professionals, providing a decentralised and accessible service. Tertiary prevention includes the development of person centered services, case management is a core component which ensures patients’ needs are met (e.g. childcare, financial or vocational assistance). The program uses an individualised for of psychotherapy (Personal and Strategic Coping Therapy) which takes into account the patient's beliefs regarding his symptoms, coming to an understanding between the therapist and patient and working out coping strategies (similar to SDM). |
| Crabtree. 2005 | Malaysia | Peer reviewed literature | Journal article | Primary (exploratory) research with a focus on/findings relating to CE | Ethnography | Ethnographic study on wards, with a focus on compliance and the demonstration of resistance by service users. |
| Deva. 2004 | Malaysia | Peer reviewed literature | Journal article | Overview or review of countrywide mental health policy, legislation or systems, featuring elements or incorporating discussion of CE | N/A | A new mental health policy was approved by the ministry of health in 1998. An objective of the policy was community participation. Ensure community participation in the planning, management and evaluation of community-based activities, to ensure their ownership, pride and adoption. |
| Dewi. 2014 | Indonesia | Peer reviewed literature | Journal article | Narrative review of, or commentary on, models of care featuring CE (e.g. Shared Decision Making, Person Centered Care) | N/A | Person Centered Care (PCC): Defined as health-care providers, such as nurses, selecting and delivering interventions or treatments that are respectful of and responsive to the characteristics, needs, preferences and values of the person or individual. Patient and family participation and involvement is an important component of delivering PCC, particularly in nursing care. |
| Directorate of Mental Health service 2014 | Indonesia | Grey literature | Report | Overview or review of countrywide mental health policy, legislation or systems, featuring elements or incorporating discussion of CE | N/A |  |
| Farida, 2018 | Indonesia | Grey literature | Article | Overview or review of countrywide mental health policy, legislation or systems, featuring elements or incorporating discussion of CE | N/A | Mental health promotion and preventive education involving the community. |
| Fujiwara. 2005 | Lao | Peer reviewed literature | Journal article | Primary (exploratory) research with a focus on/findings relating to CE | Survey | Community-based research project (household survey), which aimed to raise awareness of drug abuse and facilitate community activities to prevent drug abuse. Local partners (governors and village leaders) were involved in the entire process. |
| Gajali, 2014. | Indonesia | Grey literature | Masters thesis | Evaluation (or evaluation protocol) of a program, intervention or health system featuring CE | Pre/post evaluation | Family supportive therapy toward ability of caring for patients with schizophrenia. |
| Gow, 2012 | Cambodia | Peer reviewed literature | Journal article | Narrative review of, or commentary on, models of care featuring CE (e.g. Shared Decision Making, Person Centered Care) | N/A | Describes potential treatments that may be useful in Cambodia. |
| Sari, 2009 | Indonesia | Grey literature | Masters thesis | Primary (exploratory) research with a focus on/findings relating to CE | Pre/post evaluation | Family psychoeducation for minimising burden and increasing ability to care for loved ones. |
| Susanti, 2016 | Indonesia | Grey literature | Doctoral Thesis | Evaluation (or evaluation protocol) of a program, intervention or health system featuring CE | Qualitative focus group and/or interviews | Exploratory study looking at carers expectations for involvement in mental health services as experts (e.g. in planning and in the course of implementing care). This also found data from professionals' views of why they should engage/involve carers in services |
| Irmansyah. 2009 | Indonesia | Peer reviewed literature | Journal article | Overview or review of countrywide mental health policy, legislation or systems, featuring elements or incorporating discussion of CE | N/A | Review of current legislation relating to the human rights of people with a psychiatric diagnosis. Authors identify civic engagement/involvement as 'the most important element' that will drive change: Persons with mental illness and their families must become better organised and more skilful advocates on their own behalf. They should form organisations and associations that can engage in a confident and effective manner in discussions and decision-making about mental health systems and advocate effectively for the rights of their members. The Indonesian Mental Health Association is a body has in its membership consumers, carers and mental health professionals and advocates on behalf of people with mental illness and their families. |
| Ito et al. 2012 | South East Asia | Peer reviewed literature | Journal article | Narrative review of, or commentary on, models of care featuring CE (e.g. Shared Decision Making, Person Centered Care) | N/A | Non-governmental organizations (NGOs) have set up model mental health services, and trained both health care and non-health workers in post-conflict countries, such as Cambodia and East-Timor, where all mental health resources were destroyed. Partnerships with families were important in setting up effective community care. |
| Jirapeet. 2000 | Thailand | Peer reviewed literature | Journal article | Evaluation (or evaluation protocol) of a program, intervention or health system featuring CE | Mixed methods | A 6-week empowerment programme for HIV positive mothers developed using the participatory action research paradigm. Programme aimed to improve quality of life and psychosocial outcomes, including emotional wellbeing. Group met weekly and worked with research team to identify their needs, design action plans and implement and evaluate their actions. Researchers acted as facilitators. |
| Junardi, 2016 | Indonesia | Grey literature | Masters thesis | Evaluation (or evaluation protocol) of a program, intervention or health system featuring CE | Mixed methods | Community mental health model, involving community volunteers. |
| Helena, et al, (2013) | Indonesia | Grey literature | Report | Evaluation (or evaluation protocol) of a program, intervention or health system featuring CE | Mixed methods | Evaluation of how community mental health models can engage people with mental Illness in Indonesia |
| Kertchok. 2011 | Thailand | Peer reviewed literature | Journal article | Primary (exploratory) research with a focus on/findings relating to CE | Qualitative focus group and/or interviews | Study explores work nurses do with families of people with schizophrenia, discusses importance of involving family members in decisions about care. |
| Kusumadewi, 2016 | Indonesia | Grey literature | Masters thesis | Evaluation (or evaluation protocol) of a program, intervention or health system featuring CE | Qualitative focus group and/or interviews | Group intervention involving family members. |
| Lestari, 2009 | Indonesia | Grey literature | Masters thesis | Evaluation (or evaluation protocol) of a program, intervention or health system featuring CE | Pre/post evaluation | Group intervention involving family members. |
| Li, 2014 | Singapore | Peer reviewed literature | Conference abstract | Evaluation (or evaluation protocol) of a program, intervention or health system featuring CE | Randomised Controlled Trial | Peer-led self management programme, developed to empower people to achieve recovery and maintain a functional life |
| Li, 2013 | Singapore | Peer reviewed literature | Conference abstract | Evaluation (or evaluation protocol) of a program, intervention or health system featuring CE | Randomised Controlled Trial | Peer-led self management programme, developed to empower people to achieve recovery and maintain a functional life |
| Minarni et al., 2015 | Indonesia | Grey literature | Masters thesis | Primary (exploratory) research with a focus on/findings relating to CE | Qualitative focus group and/or interviews | Explores how can work alongside family members, to support them with managing loved one’s medication. |
| Muhlisin, 2015 | Indonesia | Grey literature | Article | Evaluation (or evaluation protocol) of a program, intervention or health system featuring CE | Pre/post evaluation | Community members were trained to help outpatients in the village. |
| Nemi et al. 2010 | Vietnam | Peer reviewed literature | Journal article | Primary (exploratory) research with a focus on/findings relating to CE | Qualitative focus group and/or interviews | Notes that no official consumer or family associations exist for the mentally ill, though recently, a group of families with autistic children in Hanoi started to work together, exemplifying a trend of establishing civil organizations on mental health care. Mentions a Community Based Mental Health Project which involves the training of local staff in mental health, including health workers, social workers and members of the women’s unions. |
| Ng et al. 2013. | Malaysia | Peer reviewed literature | Journal article | Description of the features and implementation of a specific program or intervention incorporating elements of CE | N/A | Review of patient involvement in healthcare decision-making and adopts the SDM model, defined as follows: in which patients and doctors share information and values, and patients play an active role in making healthcare decisions |
| Niman, 2013 | Indonesia | Grey literature | Masters thesis | Evaluation (or evaluation protocol) of a program, intervention or health system featuring CE | Survey |  |
| Paratharayril, 2010 | Myanmar | Peer reviewed literature | Journal article | Description of the features and implementation of a specific program or intervention incorporating elements of CE | N/A | Support local faith based organisations affiliated to Buddhism, Christianity and Islam to organise community rituals, bereavement support and burial in Burma/Myanmar. These activities were part of the early phase relief programmes (following a cyclone).   COMMUNITY KITCHEN: Everyone had a role in the community kitchen. While women and young girls helped to prepare the food, men did the cooking. Children and elders helped with arranging dining areas and feeding domestic animals. Young men helped with collecting water, wood and shopping for food items. In addition to the cooking of meals, community members also organised activities like: singing, celebration of local festivals like full moon day (an auspicious occasion in Buddhist tradition), friendship football matches and other sports activities. This initiative was owned by the community. The community gathering also provided opportunities to share their significant experiences. Those community members would otherwise have not had many other opportunities to discuss these issues and to get mutual group support.  FOCUSSED TRAUMA CARE: The aim of the programme was to help those people traumatised by the Nargis cyclone and to help them accept the realities of the aftermath and to ensure a durable and complete healing. Caregivers were trained by experienced Burmese trainers. The training included both international methods, such as psychological ¢rst aid, basic needs assessment, listening skills and group work, as well as Myanmar Buddhist meditation methods. Internationally trained trainers supervised these caregivers and facilitated their refresher training and debriefing. At first, healers met with the village heads and elders, and made home visits. They explained the purpose of the visit. For the initial rapport building, they identified a public place like a monastery, school or video hall for organising community and group activities. They organised different community events, like: story telling, action songs, drawing pictures with children and public discussions. They also provided food and recreational materials for the children and adults to get them involved in some of the community focused psychosocial activities. |
| Por and Sharharom, 2017 | Malaysia | Peer reviewed literature | Book Chapter |  | N/A | N/A - description of development of mental health services in Malaysia. |
| Rahayu, 2014 | Indonesia | Grey literature | Masters thesis | Primary (exploratory) research with a focus on/findings relating to CE | Qualitative focus group and/or interviews | Psyco-education was conducted to examine the relation between medication adherence and family support |
| Rathod et al., 2017 | South East Asia | Peer reviewed literature | Journal article | Overview or review of countrywide mental health policy, legislation or systems, featuring elements or incorporating discussion of CE | N/A | N/A - opinion piece of MH service provision in LMICs |
| Rawtaer et al, 2014 | Singapore | Peer reviewed literature | Conference abstract | Evaluation (or evaluation protocol) of a program, intervention or health system featuring CE | Pre/post evaluation | A team from the National University of Singapore collaborated with voluntary organisations to establish a psychogeriatric training and research centre in the community, focusing on prevention and early intervention for dementia, depression and anxiety. Intervention groups include Tai Chi exercise (TCE), art therapy (AT), mindfulness awareness practice (MAP) and music reminiscence therapy (MRT). In the initial 12 weeks, participants attended a single intervention of their choice weekly. For the remaining 40 weeks, as participants were keen to try other interventions, the programme evolved to tailor to this preference; participants attended a programme involving all 4 interventions. |
| Ricci et al., 2004 | South East Asia | Peer reviewed literature | Journal article | Overview or review of countrywide mental health policy, legislation or systems, featuring elements or incorporating discussion of CE | N/A | N/A |
| Shinfuku, 1998 | South East Asia | Peer reviewed literature | Journal article | Overview or review of countrywide mental health policy, legislation or systems, featuring elements or incorporating discussion of CE | N/A | The National Mental Hospital in Manila, the Philippines, once had more than 7000 inpatients. They succeeded in reducing the number to 3000 by introducing a programme called `Acute crisis intervention services (ACIS)'. The programme requested family members to stay with the patients for evaluation and intensive treatment for 3 days before they were to be admitted to the hospital. |
| Sin and Ng, 2011 | Singapore | Peer reviewed literature | Journal article | Description of the features and implementation of a specific program or intervention incorporating elements of CE | N/A | In April 207, the Community Psychogeriatric Programe (CPGP) was set up in Changi General Hospital to provide community mental health services for early detection and treatment of psychogeriatric disorders in the eastern sector of Singapore. Focuses on collaborating with care givers to meet the needs of elderly. |
| Somasundaram et al., 1999 | Cambodia | Peer reviewed literature | Journal article | Overview or review of countrywide mental health policy, legislation or systems, featuring elements or incorporating discussion of CE | N/A | Used anthropology to guide the design of health services and to implement a community mental health approach. A core group (CG) of 12 Cambodians was trained, in the theory and practice of community mental health, by an expatriate multidisciplinary team with relevant experience in Cambodia. The CG of trainers then started interventions at the individual, family and community level, and trained villagers with special positions of responsibility in their communities, governmental and NGO workers to deal with community psychosocial and mental health issues. The villagers were people such as monks, members of pagoda- committees, village chiefs, elders, leaders, monks, nuns, achaas (learned religious persons), village development committee (VDC) members, primary health care workers and school teachers. Interventions included traditional methods (e.g. massage) |
| Somasundraram & van de Put, 1999 | Cambodia | Peer reviewed literature | Journal article | Overview or review of countrywide mental health policy, legislation or systems, featuring elements or incorporating discussion of CE | N/A | Transcultural Psychosocial Organization (TPO), a nongovernmental organization has become involved in a process in which various bodies engaged in development work were invited to promote their projects in villages. Under a UNDP rehabilitation and regeneration project, 45 remote villages were allocated funds and the inhabitants were asked to select matters requiring priority attention in education, transport, agriculture, health, social affairs and other areas. TPO arranged for all the villages to be visited in three months by a core group of Cambodians who had been trained as trainers in community mental health. In all but three of the villages the people considered that dealing with psychosocial problems was the most important matter needing to be dealt with in the social affairs category.TPO has also been involved in setting up model mental health services in Battambang Pro- vince. A community-based approach was adopted in which grass-roots workers were trained so that they would be able to tackle most basic problems of mental health in the villages. A referral system was set up to tackle the more rare serious neuropsychiatric disorders. Once stabilized and perhaps on maintenance treatment, patients were referred back to community workers and local health centres for follow-up and rehabilitation. |
| Strasser, et al., 2015 | Cambodia | Grey literature | Report | Primary (exploratory) research with a focus on/findings relating to CE | Qualitative focus group and/or interviews | Explorations of the impacts of violence and coping strategies of war survivors |
| Stratford et al., 2014 | Indonesia | Peer reviewed literature | Journal article | Description of the features and implementation of a specific program or intervention incorporating elements of CE | N/A | MoSA is adopting a mental health recovery approach, prompted significantly by advocacy from the consumer and carer organisation, Perhimpunan Jiwa Sehat (the Indonesian Mental Health Association), and collaboration with Australian organisations Mind Australia (Mind) and Asia Australia Mental Health (AAMH). The program focuses on strong stakeholder engagement and connections. The recovery approach utilises individual, family, community and environmental resources as well as scarce specialised mental health resources. Community leaders, service users and their families have been involved since the inception of the programme.  Using a methodology called ‘Participatory rural appraisal’ to maximise community involvement, the Ministry of Social Affairs identifies key community leaders for its projects and involves them from the outset in planning and implementation. Community leaders may be village heads or chiefs of women’s organisations or community organisations, depending on whom community members select.  The Centre’s programme is divided into three phases. The initial stage runs for about six months, during which the service users are looked after and supported more closely. Once they have been socialised into the centre, they move to the second-stage recovery area for anything up to a year. Finally, they progress to the leaving preparation area, where they consolidate their recovery before heading home. Families are contacted and encouraged to be involved in this process. |
| Susanty et al., 2016 | Indonesia | Peer reviewed literature | Journal article | Evaluation (or evaluation protocol) of a program, intervention or health system featuring CE | Randomised Controlled Trial | The classroom based intervention was facilitated by local community volunteers, who were mostly formal school teachers and Sunday school teachers. Three layers: 1st prevent problems in at risk populations, 2nd interventions targeted at sub groups at risk of developing MH problems and third interventions that targeted treatment of sub groups with severe mental health problems (e.g. psychosocial counselling aimed at reducing severe psychological distress, suicidal risk and other high risk behaviours, or low intensity treatment) |
| Hernawaty,2009 | Indonesia | Grey literature | Masters thesis | Primary (exploratory) research with a focus on/findings relating to CE | Pre/post evaluation | Family supportive therapy for increasing ability of caring for patients with mental illness |
| Tol et al., 2008 | Indonesia | Peer reviewed literature | Journal article | Evaluation (or evaluation protocol) of a program, intervention or health system featuring CE | Randomised Controlled Trial | The intervention consisted of 15 sessions with groups of about 15 children over 5 weeks of a manualized classroom-based intervention. Interventionists, who had to be at least 18 years and have had at least a high school education, were selected from local target communities, based on a selection procedure assessing social skills through role-plays. Once selected, interventionists received a 2-week training program. They were generally people with no formal mental health training but had some experience as volunteers in humanitarian programs |
| Visanuythin et al., 2006 | Thailand | Peer reviewed literature | Journal article | Description of the features and implementation of a specific program or intervention incorporating elements of CE | N/A | Co-ordinated response to Tsunami. The village or community leaders were asked and encouraged to continue their role. Teachers and community leaders were used as conduits for information and discussions were held in 140 schools. Each village identified a number of volunteers who were responsible for looking after up to 10 families. These volunteers were trained to provide knowledge on MH issues especially as a result of a disaster, assessment methods to identify those suffering and increased awareness of service provision. They were encouraged to build buddy support systems and use statutory infrastructure to gain support. |
| Wardaningsih, S. 2018 | Indonesia | Grey literature | Article | Description of the features and implementation of a specific program or intervention incorporating elements of CE | N/A | Giving Information about mental health, mental illness, how to treat patients. Some cases were giving home visit by health workers, and evaluation. |
| Wardaningsih, 2007 | Indonesia | Grey literature | Masters thesis | Evaluation (or evaluation protocol) of a program, intervention or health system featuring CE | Pre/post evaluation | Family psycho-education for minimising burden and increasing ability for caring for people with mental illnesses. |
| WHO 2003 | Indonesia | Grey literature | Report | Overview or review of countrywide mental health policy, legislation or systems, featuring elements or incorporating discussion of CE | N/A | Strategic Intervention by health workers should accommodate community perspective. Community engagement is needed for every phase of disaster management. |
| Wig et al., 1980 | Philippines | Peer reviewed literature | Journal article | Primary (exploratory) research with a focus on/findings relating to CE | Mixed methods | Used research with service users and carers to inform interventions. |
| Wiyati, 2009 | Indonesia | Grey literature | Masters thesis | Evaluation (or evaluation protocol) of a program, intervention or health system featuring CE | Pre/post evaluation | Family psycho-education for increasing ability of caring for patients with social isolation. |
| Wong et al., 2017 | Singapore | Peer reviewed literature | Journal article | Evaluation (or evaluation protocol) of a program, intervention or health system featuring CE | Qualitative focus group and/or interviews | Case management involving caregivers, with the aim to raise awareness of and reduce stigma associated with psychosis, establish links with primary healthcare providers and collaborate in the detection, referral and management of those with psychosis and improve the outcome of its clients and reduce the burden of care on their families. |
| Yeo Chen Kuan et al., 2018 | Singapore | Peer reviewed literature | Journal article | Evaluation (or evaluation protocol) of a program, intervention or health system featuring CE | Mixed methods | Intervention designed to increase carer involvement in case management of service users. Intervention designed following interviews with case managers. Incorporated carers preferences for involvement into the intervention. Carers encouraged to attend meetings and called if not able to attend. |
